# “I will not stop visiting!” a qualitative study of community health workers’ reluctance to withdraw household support following the end of a community-based intervention in Zimbabwe

**DOI:** 10.1186/s12913-018-3531-x

**Published:** 2018-09-17

**Authors:** Joanna Busza, Ethel Dauya, Memory Makamba, Rashida A. Ferrand

**Affiliations:** 1 Department of Public Health, Environment and Society and Centre for Evaluation, London School of Hygiene and Tropical Medicine, Keppel Street, London, WC1E 7HT UK; 2 Biomedical Research & Training Institute, No. 10 Seagrave road, Avondale, Harare, Zimbabwe; 3 Department of Clinical Research, London School of Hygiene and Tropical Medicine, Keppel Street, London, WC1E 7HT UK

**Keywords:** Zimbabwe, Community health workers, Intervention, Social support, Children, Caregivers, Qualitative research, HIV/AIDS

## Abstract

**Background:**

Community Health Worker (CHW) programmes are increasingly important in HIV service delivery. CHWs’ familiarity with the local context can improve intervention acceptability and sustainability but concerns have been raised about potential exploitation and “burnout” of CHWs as they become emotionally involved in clients’ lives. Little attention has been paid to what happens at the end of time-limited CHW interventions. This study aimed to examine the experience of CHWs’ *withdrawal* from clients and their families.

**Methods:**

We conducted a qualitative study of CHWs’ experiences of “exiting” from households during the ZENITH (Zimbabwe Study for Enhancing Testing and Improving Treatment of HIV in Children) intervention, which provided 12 structured home visits over 72 weeks to families with children recently diagnosed with HIV. We conducted semi-structured interviews at 12 and 18 months with all 19 CHWs delivering the intervention and 36 purposively selected caregivers who received home visits. Analysis focused on perceptions of the end of the trial, when CHWs completed the scheduled home-based visits and there was no guarantee of programme continuation beyond the study.

**Results:**

Termination of scheduled home visits caused significant distress to both CHWs and the households they visited. We identify 3 thematic “lessons learned” for CHW programmes. First, CHWs derived pride and self-worth from emotional labour as they became integral to families’ improved ability to cope, motivating them to go beyond formal job requirements. Second, clients’ growing dependence on CHWs led to “exit” being interpreted as abandonment by both CHWs and households, causing distress on both sides. Finally, in response to anxiety about “abandoning” families, CHWs maintained contact with families long after scheduled withdrawal of services.

**Conclusions:**

CHWs can forge genuine bonds with households, creating expectations of long-term engagement. On the positive side, CHW derive pride from their work, attach social responsibility to their roles, and feel personal fulfilment in supporting families. If CHWs do not disengage from interventions as planned, or become demoralised by “exits”, interventions will prove less sustainable. CHWs are often lauded for their ability to develop trust with peers, yet this willingness and ability to create enduring emotional bonds could threaten programme delivery.

## Background

Community Health Worker (CHW) programmes play an increasingly important role in HIV service delivery, particularly with the current focus on maximising population-level coverage of testing and treatment [1–3]. CHWs know the local communities in which they work, understand the cultural context, and reduce the burden on skilled health workers, and thus are seen to improve sustainability of community-based care in overstretched health systems [4–6]. CHWs perform many of the support functions necessary to a successful HIV response, including mobilising community members for regular testing, accompanying patients to clinic appointments, and providing counselling, and evidence suggests their involvement improves engagement in care and reduces treatment failure [7–10].

Widespread use of CHWs is not without controversy, however, and concerns have been raised about poor remuneration, lack of opportunities for career advancement, and potential exploitation of predominantly poor and female volunteers [11–13]. Much literature is thus devoted to identifying best practices for maintaining CHWs’ motivation, performance, and job satisfaction [14–16], and reducing overwork and “burnout” [17, 18]. CHWs risk taking on their clients’ psychological distress or becoming demoralised when they confront difficult circumstances and are unable to effect positive change [19]. Addressing the emotional demands on CHWs as they become increasingly embedded in programme beneficiaries’ lives is acknowledged as a mainstay of good CHW supervision [20].

To date, little attention has been devoted to CHWs’ withdrawal of services from clients and their families, such as in the case of fixed-term interventions (i.e. a specified number of visits or time period per household) or following the end of a research study. We use the ZENITH trial (Zimbabwe Study for Enhancing Testing and Improving Treatment of HIV in Children) as an example to highlight potential distress caused to CHWs by the termination of a home-based visit programme, and present CHWs’ perspectives on withdrawing from households they visited. The ZENITH trial was conducted in seven high density communities of Harare, Zimbabwe between 2014 and 2016. The trial randomly allocated 334 children newly diagnosed with HIV and aged 6–15 to receive standard clinical care (168), or standard care plus community-based support to their primary caregiver and household (166), delivered by CHW through home visits. After 18 months of follow up, children in the intervention arm who had initiated ART had significantly lower rates of treatment failure compared to those in standard care [21]. CHWs visited children’s homes at key stages, such as soon after they registered at the clinic, following eligibility screening for ART, and before routine clinic visits. Guided by a manual developed for the intervention during formative work [22], CHWs conducted standardised activities with caregivers to address topics shown to improve retention in care and treatment adherence. These included sessions on dealing with side effects, disclosing the child’s status to the child and close family/community members, setting reminders to give medication (ART and/or cotrimaxazole), identifying sources of social support, and assessing need for referral to available services such as food support or educational subsidies.

The ZENITH programme collaborated with a local organisation, Child Protection Society (CPS), that had been deploying CHW since 2000 for HIV counselling and home-based care. CPS identified eligible volunteers, who were selected for familiarity with local communities, previous experience, and demonstrable motivation for community work. In total, 19 (16 female, 3 male) out of 20 recruited CHW worked throughout the trial period, with an average caseload of 9 households each. They liaised with 6 local public sector clinics serving high density suburbs, to which a study nurse had been attached to provide clinical care specifically to enrolled children. CHWs checked on children’s progress, accompanied families to clinics on request, and provided general psychosocial counselling. The intervention was designed for the existing Zimbabwean public health system, without requiring significant additional resources.

The ZENITH intervention is useful for exploring CHWs’ “exiting” as it was designed to be time-limited: households received 12–15 pre-planned and structured visits over 72 weeks. This was explained to each participating family from the beginning, and the final session was explicitly devoted to the completion of home visits and discussion of sustaining the child’s engagement with health services. In this paper, we explore CHWs’ emotional involvement with their clients, perceptions of “exiting” from each household, and experiences following the end of the trial. Our aim is to contribute to the growing literature on CHW programmes and how to maximise their potential without inadvertently causing harm to those at the frontline.

## Methods

To better understand the experiences of caregivers receiving the intervention and CHWs who delivered it, we conducted semi-structured interviews three times during the intervention, at baseline, after 1 year, and at the end of the trial. All 19 CHWs were interviewed at each round, while caregivers were purposively selected to ensure diversity in age and sex of the child, relationship to child (parent, aunt/uncle, grandparent, etc), and levels of participation in the intervention and engagement with services. We interviewed 26 caregivers at midline (22 female; 4 male) and a further 10 caregivers at endline (9 female; 1 male).

A female social scientist who was not a member of the intervention team conducted all interviews in a location convenient to respondents, usually their homes. First, CHW introduced the study to caregivers and if they were willing to participate, the social scientist contacted them to arrange interviews. All participants provided written informed consent. Interviews lasted 30–60 min, were conducted in Shona, digitally recorded, and transcribed directly into English by the interviewer. English transcripts were entered into NVIVO software for thematic content analysis.

For this paper, we draw on midline and endline data collected from CHWs and caregivers, examining their experiences of “intervention exit”. While our focus is on CHWs’ experiences withdrawing from households (stopping scheduled visits), these were shaped by interactions with caregivers, who shared their own concerns and anxieties with them. At midline, topic guides addressed general perceptions of delivering or receiving the intervention, and added questions about specific components, such as adherence support, encouragement to disclose the child’s HIV status, and referring family members for testing. At endline, the following questions were added to topic guides to elicit their views on completing the intervention (Table 1).

**Table 1:** Topic Guide Questions on Intervention Completion

CHW	Caregivers
As you know, the ZENITH programme was conducted for just 2 years. Now that your participation will finish, please reflect back on the whole experience and what you think will happen next. • PROBE: Do you think the way caregivers and children manage the care and adherence in future will change?	As you know, the ZENITH programme was designed for just 2 years. Now that your participation will finish, how will you use what you have learned to continue to support the child’s care and treatment? • PROBE: Do you think your management of the child’s care will change at all? How?
What do you think will be the main challenges for the families you visited now that they will not receive the home visits?	What do you think will be the main challenges you will face in future? • PROBE: How do you plan to deal with these challenges?

We also conducted a “reflection meeting” with CHWs 11 months following the trial to determine the extent to which CHWs had contact with households after the end of the programme. Of the 19 CHW who delivered the intervention, 14 attended the “reflection meeting” (3 were unavailable, 1 had left the country, and 1 had died). A loosely structured topic guide was used to facilitate open discussion on (1) retrospective reflections on being part of the ZENITH trial, (2) how it affected CHWs’ own lives, (3) contact with households since the end of the trial, and (4) perceptions of how local communities now viewed ZENITH. The meeting was conducted in Shona by the CHW supervisor, who took detailed notes in English.

Early in the process of data familiarisation, concerns about time limitations on each household’s participation in the intervention emerged as a key theme in both CHW and caregiver interviews. “Exit issues” was added to the coding framework during analysis of midline transcripts, leading to iterative development of questions added to endline topic guides. For this paper, we reviewed data coded under “exit issues”, re-read transcripts to identify further insights into CHWs’ emotional engagement with households they visited, and coded effects on CHWs’ ability and willingness to withdraw at the end of the follow-up period. We also re-coded data to differentiate between perceptions of completing household visits as designed (following 12 scheduled visits and 72 weeks of follow-up) and feelings about the end of the trial and uncertainly about the intervention’s future. Analysis within and between codes led to identification of thematic “lessons learned” presented in this paper.

Ethical approval was obtained from the London School of Hygiene and Tropical Medicine (6305), the Medical Research Council of Zimbabwe (MRCZ/A/1676) and the Biomedical Research and Training Institute (Zimbabwe) (AP108/2012). The trial was registered with the Pan African Clinical Trials Registry, number PACTR201212000442288.

## Results

In keeping with our aim to understand reactions to CHWs’ withdrawal from households and contribute to the growing literature on best to maximise CHWs’ potential without inadvertently causing harm, we structure our results as 3 “lessons learned” from the ZENITH trial. Although grounded in the urban Zimbabwean context, these lessons are likely to hold relevance for other research/ pilot projects, raising issues that should be addressed where CHW involvement is purposively finite.

### Emotional labour can instil pride and self-worth

CHWs acknowledged their work was difficult, sometimes requiring visiting household members outside their homes or during evenings and weekends. They described their efforts as evidence they took their role seriously, fulfilling their duties even when it meant prioritising needs of client households over their own. We highlight two examples below, when CHWs felt unwell or gave up disproportionate time to ensure they met household members:

*As someone who was not feeling well, I would feel compelled to go for my visits. It’s what we would have agreed to… although I was sick I did not stop going to see a child when her time was due. [CHW#10, female, midline]*

*We were meeting at the clinic, I got there and waited but she did not come. I waited and actually I waited for hours. I stayed there for about 3, 4 h sitting there. Imagine someone who has left her home without eating anything, saying “let me go and see the client!” [CHW#6, female, midline]*

Such narratives often used language that implied self-sacrifice (“*imagine someone who has left her home without eating!*”*)*, but this was conveyed with pride rather than as a complaint. CHWs felt considerable satisfaction in their work and interpreted positive change within households as personal validation.

One CHW explained how becoming personally involved with families led to their growing reliance on her for support beyond the topics and activities of the intervention manual, such that she became their primary emotional resource. She justified her intensified involvement as contributing to caregivers’ mental health and well-being, which she considered to be likely determinants of the enrolled child’s engagement in care. Her ability to provide psychosocial and practical assistance also bolstered her own self-esteem and sense of purpose:

*At times someone will tell you all her issues, which are not connected to the reason you have gone there. She will tell us about other issues, her personal life, problems she is facing in her life. She will be sharing. …. I will have to assist; I have to counsel them because this will affect their taking of medication, and this will also affect their monitoring of the child. You are now helping them so that their mental state, their mind, will remain positive. …* ***You feel like a life saver. It’s like you are someone.*** *[CHW#6, female, midline, emphasis added]*

CHW appreciated that household members expressed their gratitude, further contributing to CHWs’ commitment and confidence. Caregivers confirmed their emotional attachment to CHW during interviews, in some cases reinforcing the image of CHWs as literal “lifesavers”.

*We are so happy because were it not for them.... if [CHWs] were not around these would have been graves, do you hear? We would not have the children. [Great aunt of boy, midline]*

*You can see that they really care. You will actually feel as if you have got your relatives. Sometimes when I am facing difficulties and I am worried … if I go to … Mrs. [CHW] and I talk, l feel like the pain has gone. I feel like the problem has gone because I feel like they are my relatives. [Aunt of boy, endline]*

This sense of pride and self-worth among CHWs persisted following the end of the trial. During the reflection meeting, they reported having gained credibility in their communities, and felt they were considered local “experts.”

*Communities look up to them now, as they are regarded differently, they are regarded as full of knowledge on HIV issues. … The CHW have gained confidence in HIV issues and this helps them improve their self-esteem. [Reflection meeting notes]*

### Withdrawing from households feels like abandonment

A consequence of the emotional intimacy reported by CHWs and caregivers is that completion of the standard package of 12 home visits was perceived to betray the closely forged relationships. Caregivers frequently referred to CHW as “almost family” or “like our relatives,” and expressed anxiety and perhaps even disbelief that someone considered kin might cut off contact. Caregivers’ distress upset CHWs, who also struggled with the idea of extricating themselves from households.

*It is so important to me, were it possible it should not be stopped. You get so affected when you hear that it is coming to an end. That is what I can say. [Aunt of girl, midline]*

*If you want to look at all the exits that we wrote, most people were crying … we can see that there are challenges because they no longer have anyone to cry to … because we were sort of like family members. [CHW#11, male, endline]*

CHWs particularly viewed the 72-week follow-up period as arbitrary and not adequately based on the needs of individual families.

*When you went to tell the parents that the 72 weeks that we worked with their child have ended, they would show pain, “ah… why don’t you stay with us? You have been helping us especially on adherence.” [CHW#3, female, endline]*

*He said, “why are you leaving?” He was the first one to tell me that it’s not possible, I should continue coming to see the child. Sometimes it was difficult to exit a child at 72 weeks. [CHW#6, female, endline]*

These anxieties were rooted in CHWs’ familiarity with households’ circumstances, and concerns for those families where they had not been able to effect sustainable change, such as those who still missed appointments or needed help remembering to give children their medication regularly. CHWs felt not all households had achieved self-sufficiency and would be able to sustain children’s care and they were therefore abandoning families in need.

*I think most people, ah! most of them will remain with huge challenges because they were used to having someone coming - let’s say if some do not understand their [appointment] dates, someone would come to say “your dates are almost due”. I think they are going to have huge challenges. [CHW#4, female, endline]*

Caregivers also felt they were losing a valuable resource on which they had come to depend, and worried about their ability to cope. The excerpt below reflects concerns by a mother who found it difficult to talk to her daughter herself, relying on the CHW to directly intervene.

*The challenges that we are now facing are like, maybe if [CHW] was still coming - like when she [child] missed [medication] last time, maybe he might have talked to her. He might have sat down and counselled her. … So maybe it would help if she were getting support from people like [CHW] because she would know what the advantages are for her and what she is not supposed to be doing. … Maybe as an organization they will leave us somewhere [else] were we could get counselling [Mother of girl, endline]*

### Personal engagement outlasts project cycles

As a result of perceived abandonment, CHW did not fully comply with “exiting” from households. Although they completed the final session, they responded to caregivers’ pleas for additional contact on an informal basis.

*[I said to the CHW] “If only you could continue it will be helpful for us. A child needs to constantly see that people do care about her sickness. Therefore, they actually remember, and they actually think [about it]. … I really like what you are doing” [Aunt of girl, midline]*

*Some were hurt saying, “we are no longer meeting as the program has finished”. I said “no, we will be coming back to check on how the children are. Are you going to the clinic? … Whatever challenges you will be having you can call me”. I left them my number. [CHW#12, female, endline]*

All 14 CHWs who attended the reflection meeting reported still being in touch with households they had visited, to differing degrees. Some exchanged text message or phone calls with caregivers and/or children, others might encounter household members in passing and stop for an update, while some reported maintaining visits at their own cost. CHWs gave several overlapping and complex reasons for their difficulty “exiting”. As previously mentioned, they did not want to disappoint caregivers, nor abandon families with whom they had become close. Furthermore, they described social pressure to be good community members, and did not want to be perceived as selfish and dismissive of social bonds. In all but one case, CHWs reported that contact was initiated by caregivers looking for advice or assistance, including requests for clinic fees. As illustrated below, CHWs interpreted their ongoing involvement as part of their civic duty as individuals embedded in the local community, which outlived their role as CHWs.

*They [programme management] said “ah, close your books”. But our relationship has not ended. Some are still phoning. Like, “Mrs. [CHW] are you not coming to see the child?” Then one of these days you will have to look for your own dollar so that you can go and see this child. You will ask her, “are you taking medication? Are you going to get medication at the clinic etc?” … I will not stop visiting. [CHW#7, female, endline]*

*Are we going to divorce with them, with these children and that family? Remember we are in the community. We are within the same community with those people. Like some of the people that we go to see stay close, … we will be a family and* ***they will always be looking at me for support*** *. But after 2 years ZENITH will be saying it is over. [CHW#16, male, midline, emphasis added]*

When the intervention and trial ended, CHW did not feel emotional relationships born out of the programme could be terminated.

*I felt like we now had a bond such that even now that the research is over - we finished and we bade them farewell - but they still want me to go and see them. And I am even free to continue going to see them. ... A bond was created by the programme. [CHW#6, female, endline]*

Some of CHWs’ disillusionment related to the intervention’s delivery as part of research, and CHWs’ lack of clarity about the likelihood of the intervention being rolled-out. During the final interviews, several CHWs appeared confused about why the trial’s successful results did not guarantee intervention scale-up.

*The program has ended - ah! It is surprising, because we were thinking it would continue. When they realised that after 2 years we have done a good job, it will continue. It will remain. Therefore, we were surprised that the program has ended. [CHW#14, female, endline]*

In fact, aspects of the ZENITH intervention model were subsequently adopted by local social services and community-based organisations. At the reflection meeting, CHWs reported being formally and informally engaged in new schemes providing similar home support to caregivers of new diagnosed children:

*[Local NGO] is implementing nearly the same programme, looking after children on second line therapy. The programme is being implemented with input from CHW who helped craft visits in a similar model to ZENITH. … More children have been incorporated in the program, with the ZENITH model in mind. … Some healthcare workers from the Harare City Health Clinics liaise with CHW when they find difficulties in dealing with issues like disclosure among adolescents. They ask the CHW to help .... [Reflection meeting notes]*

## Discussion

CHWs were at the heart of the ZENITH intervention, and the trial’s success hinged on their skills and commitment. The intervention was designed to fit Zimbabwe’s overstretched health system, maximising potential for scale-up by using an existing cadre of volunteers to deliver community-based support to caregivers of children newly diagnosed HIV, a population with poor treatment outcomes [23]. We were cognisant of debates around task-shifting to CHW regarding fair remuneration, opportunities for career development, and integration into formal healthcare structures, and, as reported elsewhere [24], we designed the intervention in keeping with identified best practices for optimising CHWs’ job satisfaction, performance and sustained motivation [2].

As noted by others, we found CHWs developed close personal relationships with households [25, 26]. CHWs expressed gleaning pride and validation through emotional engagement, describing it as more uplifting than exhausting, even when their efforts to support families went beyond job requirements. CHWs gave examples of devoting long hours to the households they visited, counselling on topics unrelated to the standardised content, and maintaining scheduled visits even when unwell. CHW were rewarded by caregivers’ gratitude and evidence of positive change, contributing to their sense of self-worth. A study in Nepal also found CHW to be motivated by intrinsic rewards such as self-fulfilment and commitment to meeting moral obligations, positively influencing job satisfaction and retention [27].

Yet personal involvement with households carries risks. Pandey et al. [18] refer to “deep emotional work” by Accredited Social Health Activists (ASHA) in India, which negatively affected their ability to maintain appropriate boundaries and led to “burnout”. In our study, this deep engagement came to the fore when CHWs were meant to withdraw from households, putting their emotional needs at odds with programme requirements. CHWs and caregivers characterised their relationships as “like family,” implying intimacy and long-term involvement. As a result, both CHWs and caregivers viewed the end of home visits as abandonment, and CHWs felt they would be responsible for any subsequent regression in children’s and caregivers’ mental and physical health.

Caregivers’ anxiety further exacerbated CHWs’ concerns about terminating contact. In response, many chose to continue relationships with families in their own time and at their own expense, sustaining their emotional labour without the support previously offered by ZENITH. Although CHWs no longer had formal responsibilities after the trial, as the model was scaled-up or replicated, CHWs would be assigned new households, and thus potentially faced an ever-increasing caseload. One reason CHWs gave for their continued support to households was their “community embeddedness,” which meant relationships established during the intervention became part of local social structures and expectations of support.

Gale et al. [28] introduce the concept of “synthetic support” in their qualitative examination of CHW pregnancy support in the UK. This refers to the artificially introduced relationships with CHWs, designed to be content- and time-limited. In our study, CHWs and caregivers clearly felt that *genuine* social bonds had been created and did not easily recognise the support as “synthetic” in the sense it could be withdrawn on schedule. While there have been calls to better acknowledge the psychological consequences of CHWs’ emotional engagement [12, 18], the reality is that one of the most crucial criteria for CHW programme success is CHWs’ strong relationship with communities [29]. It may therefore prove more fruitful to proactively address the emergence of social bonds and longer-term contact, working with CHWs to develop context-appropriate strategies for creating personal boundaries and supporting CHWs with pressures of enduring expectations.

## Conclusions

One appeal of concise, time-limited structured interventions is cost effectiveness, and likely sustainability at scale. CHWs have been shown to build rapport and trust with community members and make interventions more acceptable by virtue of their origin in and familiarity with local social and cultural contexts. Our study found that CHWs derived pride from their work, attached social responsibility to their roles, and derived personal fulfilment in supporting families, all of which demonstrate the positive contribution of CHWs’ work to both households and themselves. If CHWs do not disengage from households as planned, however, or if the stress caused by having to initiate regular “exits” from families with whom they have established close relationships leads to demoralisation, then programmes will not prove feasible to deliver in the long term. There is a risk that CHWs’ willingness and ability to forge enduring emotional bonds could threaten programme delivery if expectations on both sides are not proactively managed.
